# Quantitative Assessment of the Importance of Phenotypic Plasticity in Adaptation to Climate Change in Wild Bird Populations

**DOI:** 10.1371/journal.pbio.1001605

**Published:** 2013-07-09

**Authors:** Oscar Vedder, Sandra Bouwhuis, Ben C. Sheldon

**Affiliations:** 1 Edward Grey Institute, Department of Zoology, University of Oxford, Oxford, United Kingdom; 2 Institute of Avian Research, Wilhelmshaven, Germany; Australian National University, Australia

## Abstract

Predictions about the fate of species or populations under climate change scenarios typically neglect adaptive evolution and phenotypic plasticity, the two major mechanisms by which organisms can adapt to changing local conditions. As a consequence, we have little understanding of the scope for organisms to track changing environments by *in situ* adaptation. Here, we use a detailed individual-specific long-term population study of great tits (*Parus major*) breeding in Wytham Woods, Oxford, UK to parameterise a mechanistic model and thus directly estimate the rate of environmental change to which *in situ* adaptation is possible. Using the effect of changes in early spring temperature on temporal synchrony between birds and a critical food resource, we focus in particular on the contribution of phenotypic plasticity to population persistence. Despite using conservative estimates for evolutionary and reproductive potential, our results suggest little risk of population extinction under projected local temperature change; however, this conclusion relies heavily on the extent to which phenotypic plasticity tracks the changing environment. Extrapolating the model to a broad range of life histories in birds suggests that the importance of phenotypic plasticity for adjustment to projected rates of temperature change increases with slower life histories, owing to lower evolutionary potential. Understanding the determinants and constraints on phenotypic plasticity in natural populations is thus crucial for characterising the risks that rapidly changing environments pose for the persistence of such populations.

## Abstract 2

Parameterisation of a mechanistic population model with data from a 51-year study on great tits suggests that phenotypic plasticity is crucial for viability of bird populations under current climate change scenarios.

## Author Summary

Predictions about the effect of climate change on organisms often ignore the possibility that organisms can evolve, or that they have an inbuilt capacity to cope with changing conditions. In order to understand the potential for existing populations to adapt to climate change, and the relative risks of extinction, such processes need to be modelled together with projected changes in climate. In this paper, we use data from a long-term study (51 years) of a small bird, the great tit, to model how birds can match the time they breed each year with the time their food is most abundant, and how this match can change with a changing climate. We found that evolution offers the chance for short-lived birds to adapt at the rate of climate change that is expected over the next century, but that the most important way that birds can cope with climate change is their evolved ability to adjust their behaviour depending on the environment they experience (“plasticity”). We extrapolated the model to other bird species, to estimate their relative vulnerability to changing climate. The model shows that longer-lived species (which also tend to have fewer offspring and take longer to reach sexual maturity) are more vulnerable to extinction because their evolutionary potential is lower. For such species, the importance of close adjustment to their environment becomes even greater. Hence, knowledge of the causes and limits of individual adjustment to the environment are crucial to predict the fate of populations under climate change.

## Introduction

Evidence that climate change influences many properties of wild populations of animals and plants is now ubiquitous [1]–[4]. As a consequence, there is widespread concern about the demographic and evolutionary effects of changing climate for the long-term viability of populations. A popular approach to study the impact of climate change on population viability is the use of “climate envelope models” or “niche models.” These models take environmental correlates of species presence, combined with climate change projections, to predict range shifts and extinction rates (e.g., [5]–[7]). However, such projections do not take a population's ability to adapt to changing environmental conditions into account [8]–[10]. Further, since habitat fragmentation potentially constrains range shifts to track the optimal environment, populations of many species will have to adapt *in situ* to a changing environment to avoid extinction. Such models may therefore not be ideally suited to predict sustainable rates of climate change for existing populations.

In contrast, mechanistic population models focus specifically on those population attributes that underlie population persistence. By assessing how phenotypic traits that influence population growth rate are affected by environmental variables, predictions of the fate of populations under varying rates of environmental change can be made [11],[12]. Recently, Chevin et al. [13] proposed a mechanistic population model that predicts the critical rate of environmental change that allows long-term population persistence by local adaptation. The main novelty of the model lies in the fact that it allows local adaptation by both genetic change (i.e., micro-evolution) and phenotypic plasticity (the potential for a given genotype to be expressed differently in different environments [14]). Since phenotypic plasticity is currently recognized as being responsible for the majority of adaptive phenotypic changes in response to climate change [15]–[18], this model is an important step forward in predicting effects of climate change on population persistence. The model combines demographic population properties (e.g., generation time, maximum intrinsic growth rate) with quantitative genetic measures (e.g., additive genetic variance, strength of stabilising selection on traits sensitive to climate change), and allows for phenotypic plasticity by incorporating the effect of the environment on the trait. Since the purpose of the model is to make predictions about the fate of wild populations, the required parameters should ideally also be estimated using data from those same populations. To do so may be challenging, as it requires long-term data describing responses to the environment, as well as extensive pedigree and fitness data, a combination of information typically only found in long-term studies of marked individuals [19].

A long-term population study on great tits (*Parus major*) breeding in Wytham Woods near Oxford (UK) offers a rare opportunity to parameterise the model of Chevin et al. [13] for a single population, and hence to investigate the projected effects of climate change on population viability allowing for plasticity and evolution. For many wild bird species—both marine and terrestrial species—reproduction is restricted to a short annual period, in which there is sufficient food available to meet the needs of offspring production. This period varies annually and is set by the responses of lower trophic levels to abiotic factors, which are ultimately shaped to maximise productivity [20],[21]. Although timing of this period is sensitive to ambient temperature, there is no *a priori* expectation that different trophic levels respond similarly to change in temperature. Hence, climate change has the potential to upset synchrony between food availability and timing of reproduction in birds, which may have important consequences for population viability [20],[21].

Successful reproduction in great tits depends to a large extent on synchronization of offspring food demand with a brief annual peak in caterpillar abundance. This can be achieved by individual adjustment of laying date to early spring temperature, which predicts the timing of the peak in food availability [22]. Repeated observation of females breeding in multiple years yields observations of individual laying dates under different spring temperatures, providing a measure of phenotypic plasticity, or the “reaction norm” to temperature [23],[24]. In addition, long-term monitoring of the annual timing of peak abundance of caterpillars feeding on newly emerged pedunculate oak (*Quercus robur*) leaves provides an estimate of how the optimal great tit laying date changes with temperature. An estimate of the optimum derived from an independent aspect of the environment is preferable to one derived from direct observations of birds, as it is unaffected by a potential constitutive cost of plasticity or differences in intrinsic individual quality of birds with different laying dates.

Here we parameterise Chevin et al.'s [13] model with estimates from the long-term study of Wytham Woods' great tits, and so calculate the maximum rate of sustained change in early spring temperature that allows long-term persistence of this population. We also use the model to explore the dependence of population persistence on currently observed phenotypic plasticity, and further to explore the interactions between life-history variation and plasticity as a key element in persistence of populations facing environmental change. Our aim was thus to use the model as an heuristic tool to understand the importance of phenotypic plasticity in adaptation to climate change.

## Results

### Impact of the Environment

**Figure 1:**
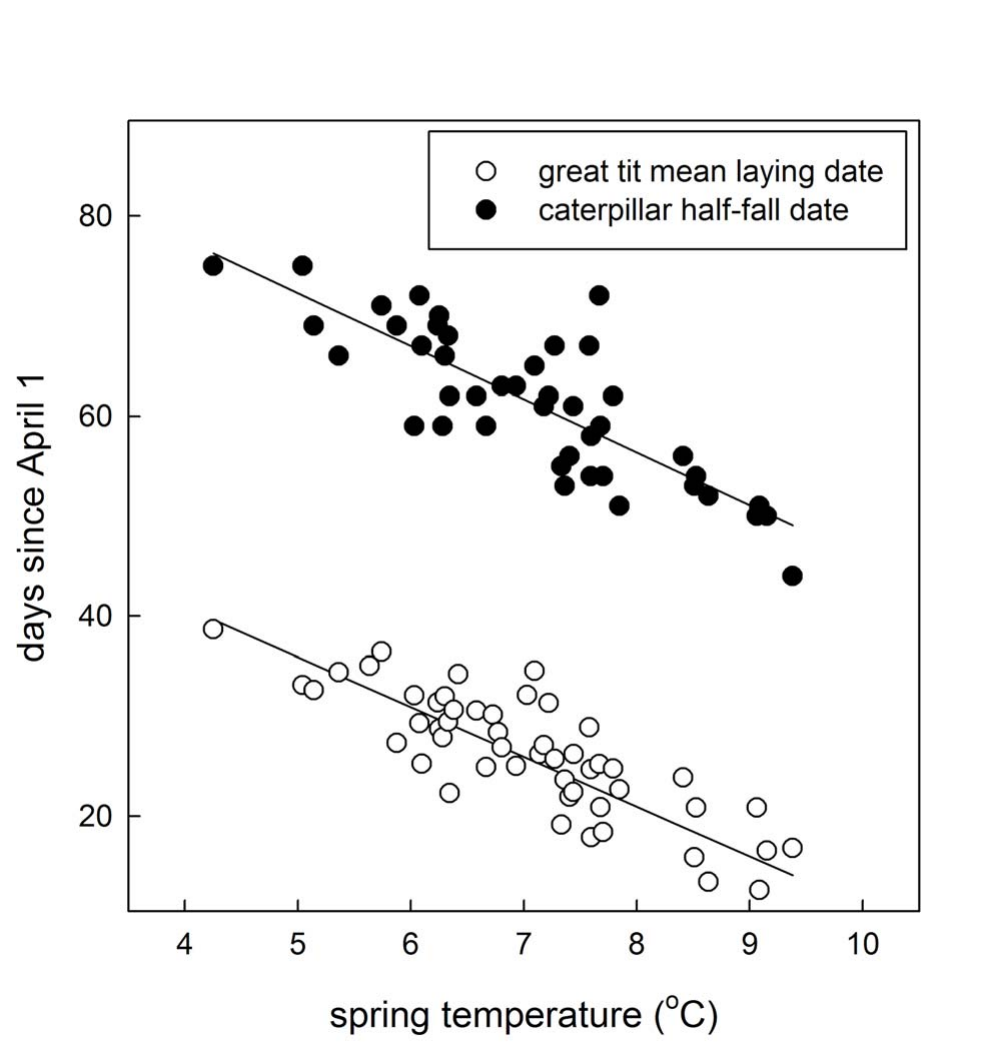
Annual mean great tit laying date and annual caterpillar half-fall date in Wytham Woods plotted against spring temperature. The trend line for laying date represents the average within-individual response to spring temperature (see Results). The trend line for half-fall date represents the average response to spring temperature.

Inter-annual changes in the spring temperature experienced by individuals had, as expected, a pronounced effect on great tit laying date (χ*^2^* = 101.25; Δ*df* = 1; *p*<0.001) with individual females laying an estimated 4.98 (±0.49 standard error [SE]) days earlier for each 1°C rise in spring temperature (Figure 1). The within-individual response to spring temperature was similar to the difference in laying date between individuals that experienced different spring temperature, as averaged over all their reproductive attempts (estimate ± SE = −4.31±0.50; χ*^2^* = 75.39; Δ*df* = 1; *p*<0.001), indicating that the relationship between annual population average laying date and spring temperature is predominantly caused by phenotypic plasticity (Figure 1), as found previously [22]; note that any evolutionary response to selection would be captured in the between-individual term. Phenotypic plasticity in response to increasing mean spring temperature has resulted in an advance of average laying date by about 2 wk in the last half century [22].

Caterpillar half-fall date (an index for timing of peak food availability; see “Materials and Methods”) also reacted strongly to spring temperature (χ*^2^* = 90.10; Δ*df* = 1; *p*<0.001), with half-fall date advancing an estimated 5.30 (±0.56 SE) days per 1°C rise in spring temperature (Figure 1), a rate only slightly more rapid than the response of great tits over the same period. The effect of spring temperature on half-fall date did not change over time (spring temperature×year; estimate ± SE = −0.05±0.04; χ*^2^* = 1.57; Δ*df* = 1; *p* = 0.21), and we thus assume that the reliability of spring temperature as a cue for the optimal laying date has been constant. Overall we conclude that the response in laying date of individual great tits to spring temperature (corresponding to *b* in Chevin et al.'s model; see Table 1) closely matches the optimal response (the term represented by *B* in their model).

**Table 1:** Summary of parameter estimates, obtained from the long-term population study on great tits in Wytham Woods, required to estimate the critical rate of temperature change (*η*_c_), following the model by Chevin et al. [13].

Parameter	Description	Estimate (SE)	Source
*σ^2^h^2^*	Additive genetic variance in laying date	2.62 (0.67)	[31]
*γ*	Strength of annual stabilising selection on laying date (based on number of recruits)	0.0061 (0.0010)	This paper
*T*	Generation time (average female age at reproduction, in years)	1.81 (0.01)	[51]
*r_max_*	Maximum intrinsic rate of annual population growth	0.49	[28]
*B*	Environmental sensitivity of selection (slope of annual caterpillar half-fall date against temperature)	−5.30 (0.56)	This paper
*b*	Phenotypic plasticity (average individual slope of laying date against temperature)	−4.98 (0.50)	This paper

See Text S1 for more details on parameter estimation.

### Probability of Population Extinction

**Figure 2:**
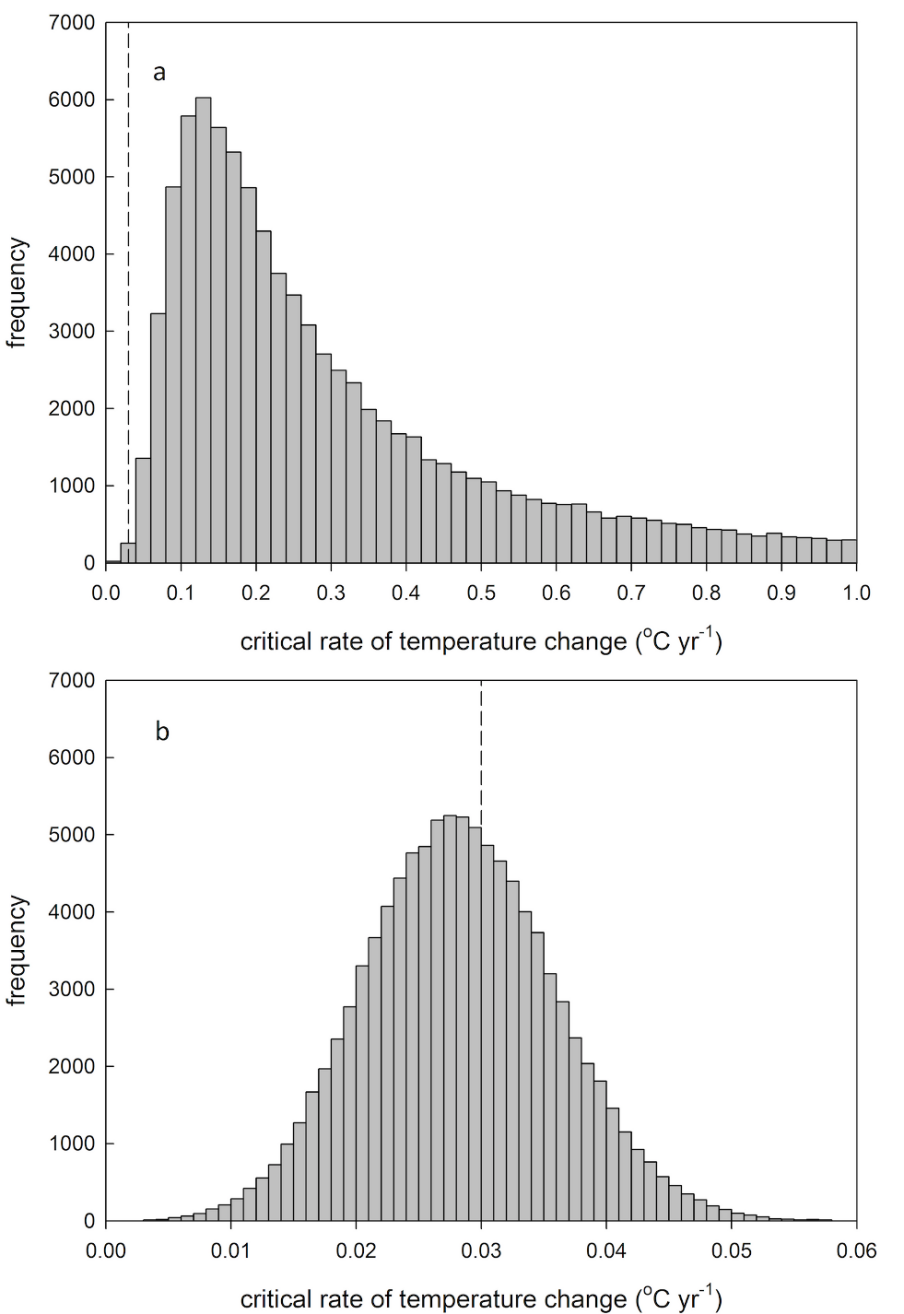
Frequency distribution of model predicted critical rate of temperature change that allows population persistence (*η*_c_) , (**a**) based on 100,000 simulations randomly sampling from error distributions of parameters *σ^2^h^2^*, *γ*, *T*, *B* and *b*, estimated for great tits in Wytham Woods (see Table 1). The dashed vertical line represents the predicted rate of local temperature change, under the high emissions scenario [25]. Note that the distribution is highly skewed, with the modal outcome being biased to lower values of *η*_c_ and a long tail of high values of *η*_c_ (for visual purposes the *x*-axis was cut off at *η*_c_ = 1.0). This is because the difference between the reaction norm of great tit laying date in response to spring temperature (*b*) and the optimal reaction norm (*B*) is modelled in absolute terms while in the simulations *b* often exceeds *B*, causing the average outcome of |*B−b*| to be higher than the outcome for the point estimate. (b) Frequency distribution of *η*_c_, based on 100,000 simulations randomly sampling from error distributions of parameters *σ^2^h^2^*, *γ*, and *T*, assuming there is no phenotypic plasticity (for this population, |*B−b*| = 5.30). The dashed vertical line represents the predicted rate of local temperature change, under the high emissions scenario [25]. Note that the scale on the *x*-axis differs between the two figures.

Combining parameter estimates for Chevin et al.'s model (Table 1), the Wytham great tit population is predicted to be able to adapt to a maximum long-term rate of increase in spring temperature of 0.47°C y^−1^, i.e. >15 times the rate of temperature change of 0.030°C y^−1^ predicted under a high emissions scenario for this location and time in the annual cycle [25]. However, this estimate does not take uncertainty in parameter estimates into account. To calculate the probability that the modelled critical rate of change (*η*_c_) will fall below 0.030°C y^−1^ while accounting for parameter uncertainty, we ran 100,000 simulations, with each simulation randomly sampling from a normal error distribution of parameters *σ^2^h^2^*, *γ*, *T*, *B*, and *b*. This resulted in an estimated probability of 0.001 that *η*_c_ falls below 0.030°C y^−1^ (Figure 2a), and hence again very little likelihood of extinction due to predicted temperature change. If we assume that there is no phenotypic plasticity in great tit laying date (hence: |*B−b*| = 5.30) the point estimate of *η*_c_ is 0.028°C y^−1^, with a 60% probability of population extinction (*η*_c_<0.030) when the error around the parameter estimates of *σ^2^h^2^*, *γ*, and *T* is taken into account (Figure 2b). Hence, the likelihood of population persistence in a changing environment depends heavily on the presence of phenotypic plasticity, as extinction risk is >500-fold higher in the absence of phenotypic plasticity.

### Phenotypic Plasticity and Life-History Variation

**Figure 3:**
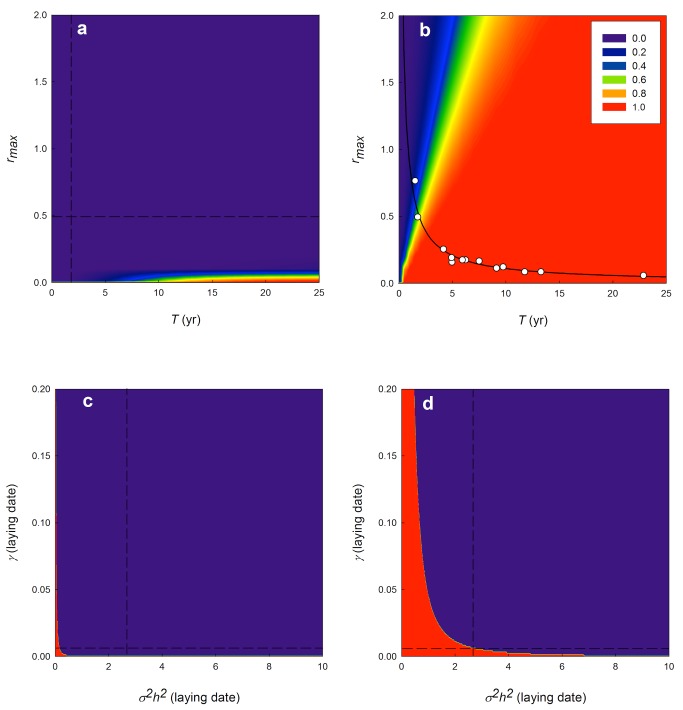
Probability of population extinction (*η*_c_<0.030), based on 100,000 simulations incorporating the error distribution of parameter estimates, plotted for (a) *r_max_* and *T*, assuming the observed phenotypic plasticity (|*B−b*| = 0.32), (b) *r_max_* and *T*, assuming there is no phenotypic plasticity (|*B−b*| = 5.30), (c) *γ* and *σ^2^h^2^* (for laying date) assuming the observed phenotypic plasticity (|*B−b*| = 0.32), (d) *γ* and *σ^2^h^2^* (for laying date) assuming no phenotypic plasticity (|*B−b*| = 5.30). Open circles and solid trend line in (b) represent estimates for *r_max_* and *T* of 13 bird species, and their derived interrelationship (*r_max_* = 0.92*T* ^−0.92^), illustrating a general life-history pattern in birds [28]. The dashed lines in (a, c, and d) represent the estimated values for the parameter on the axis, for Wytham Woods' great tits (see Table 1).

We explored the sensitivity of the probability of population extinction for other species with different life histories, assuming similar rates of change in the environment (see Discussion), by varying the demographic and life-history parameters *T* (generation time) and *r_max_* (maximum rate of annual population growth) while holding other parameters in the model constant; these effects are illustrated with contour plots in Figure 3a and 3b. This exercise revealed that with a difference in observed and optimal reaction norm equivalent to that seen in Wytham great tits (|*B−b*| = 0.32), which we take as indicative of a population showing close matching to the environment (note that, when |*B−b*| = 0 [perfect tracking of the environment], *η*_c_ is undefined), the model suggests little concern about a population being unable adapt to a rate of environmental change equivalent to an increase in spring temperature of 0.030°C y^−1^, over most of the range of *T* and *r_max_* (Figure 3a). However, since the fundamental life-history trade-off between survival and reproduction leads, in general, to a negative correlation between *T* and *r_max_* [26],[27], organisms with the slowest life histories (i.e., high *T*, low *r_max_*) are, even with quite close phenotypic matching (Figure 1), not far from the region at which risk begins to be appreciable.

**Figure 4:**
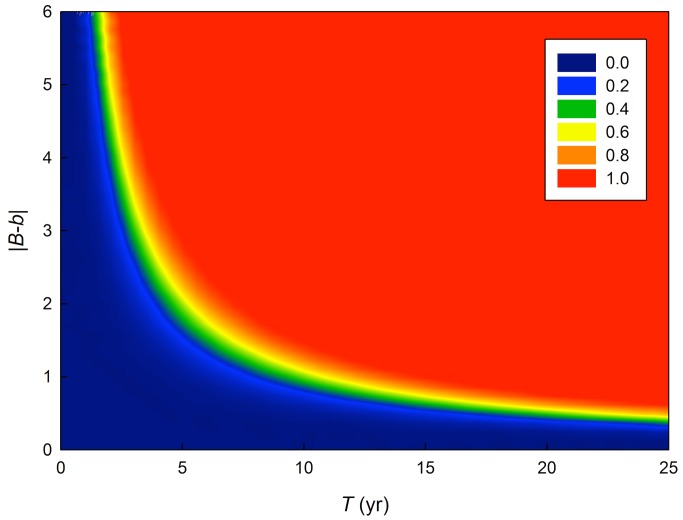
Probability of population extinction (*η*_c_<0.030) plotted for generation time (*T*) and the mismatch between the observed and optimal phenotypic response to temperature (|*B−b*|), assuming *r_max_* = 0.92*T* ^−0.92^ and incorporating the error distribution of *γ* and *σ^2^h^2^* as estimated for Wytham Woods' great tits.

It is not plausible that great tit life history parameters such as generation time would evolve rapidly enough to the extent that the risk of population extinction would become substantial with the observed phenotypic plasticity. However, by setting phenotypic plasticity to zero (|*B−b*| = 5.30), we can explore the importance of phenotypic plasticity, and the extinction risk given these rates of environmental change, across the life-history continuum for other birds. Plotting *T* and *r_max_* values for 13 species of birds [28] in Figure 3b shows a general pattern (*r_max_* = 0.92*T* ^−0.92^) under which populations of other species with longer generation times are much less likely to adapt to increasing temperatures in the absence of phenotypic plasticity, assuming that the quantitative genetic parameters determining evolvability (*σ^2^h^2^* and *γ*) are similar to that of the studied population of great tits (see also Figure 4).

### Sensitivity to Changes in Evolvability

We then explored the sensitivity of our conclusions to varying evolvability of populations while holding other quantities constant. Figure 3c shows that, with the observed life history and phenotypic plasticity in laying date, our conclusions about the ability of this great tit population to adjust to the high emissions scenario projected temperature change of 0.030°C y^−1^ are quite robust to variation in the estimated genetic variance (*σ^2^h^2^*) in laying date and strength of stabilising selection (*γ*) on laying date. In the absence of phenotypic plasticity, the population is at the threshold at which the additive genetic variance (*σ^2^h^2^*) in laying date is insufficient for the population to remain viable (Figure 3d). Equally, if the strength of stabilising selection on the match with the environment were weaker, extinction risk would also be elevated. However, in general it appears that a relatively fast life history provides sufficient potential to considerably reduce the risk of population extinction due to climate change.

## Discussion

In this study we explored the viability of a well-studied wild bird population to changes in climate predicted to the end of this century, by parameterising a mechanistic model by Chevin et al. [13]. We further explored the dependence of population viability on phenotypic plasticity as a form of adaptation to the environment, and the extent to which these conclusions depend on life history, and on evolvability. Our general conclusions are that the importance of phenotypic plasticity in adaptation to climate change is strongly dependent on life history. Short-lived species, with high reproductive rates, are more resilient to expected rates of climate change even with relatively little phenotypic plasticity, and while phenotypic plasticity is likely to be an adaptive response to environmental uncertainty in such species, it is not the only potential form of adaptation to climate change unless generation time encompasses multiple years and the rate of reproduction is slow. While the parameters we fitted to the model were determined by the specific details of our study system, we discuss below the extent to which our conclusions can be generalised.

Like all models, the model by Chevin et al. [13] makes assumptions to simplify reality. For example, the model assumes no stochastic variation in optimal timing of reproduction. Stochastic variation occurring over time scales shorter than a species' generation time can only be countered with phenotypic plasticity, and as such the model may underestimate the importance of phenotypic plasticity. Our conclusions should therefore be interpreted with respect to long-term directional climate change only, assuming that population demography is buffered against environmental stochasticity. Such buffering, in the present system, may be accomplished by the fact that generations overlap and adult survival is largely independent of the match with the environment [29],[30]. This possibility is not accounted for by the model as it assumes non-overlapping generations. Further, if adult survival is independent of the match to the environment, any evolutionary response to directional change is likely to be retarded. Moreover, in applying the model we have assumed that both the response to environmental cues and the dependence of the environment on climate can be extrapolated outside the ranges currently observed. In the case of the three trophic levels studied here (oaks, caterpillars, and great tits) the possibility remains that they exhibit differential phenotypic responses or physiological tolerances to increased temperature. If so, it is questionable whether the degree of matching can be assumed to be fixed over time. In this respect it is noteworthy that the model also allows for overcompensation, which would be just as detrimental as under-compensation, and causes a modification of predictions when parameter error is incorporated, as this results in a skewed error distribution of |*B−b*| (Figure 2a).

Although we incorporated error in parameter estimates for our predictions of extinction probability, this does not exclude the possibility that certain parameters and associated errors are systematically over- or underestimated. Estimates of the additive genetic variance for laying date in birds have been derived in several ways, from different study species with a range of life histories (reviewed in [31]; see also Text S1 for further discussion). While there is considerable variability in the estimates, it is likely that many estimates are inflated by a failure to control effectively for common environmental effects between relatives, which can be expected to be considerable for a trait with a strong link to environmentally determined phenology (see also [32],[33]). In this study we used an estimate of *σ^2^h^2^* derived from a very low heritability estimate (0.03) from an animal model controlling for several types of environmental variance [31]. We suspect that estimates of the additive genetic variance for time of breeding will be closer to this value than many previous estimates once appropriate environmental control is built into models. Sex-limited expression of traits will reduce the response to selection. While laying date is a phenotype only expressed by pairs of birds, in many, but not all, species it is primarily determined by the female [34]. Hence, the predicted evolutionary response to selection can be over-estimated if sex-limitation is not considered. The strength of stabilising selection on timing of breeding used here (*γ*) is more likely to be an underestimate as this is based on observational data at the level of the population. Two likely additional sources of stabilising selection that are not considered by such data result from, first, the extent to which individuals optimise timing of breeding to the phenology of their local environment. If there are different optima for different locations, then birds in the tails of the population phenotypic distribution may be closer to their local optimum than assumed: hence phenotypes should be measured at the appropriate relative scale. A second effect that will underestimate stabilising selection is the extent to which apparent directional selection on laying date results from phenotypic covariance between other aspects of individual quality and breeding date [35]. Figure 3d suggests that, if the match between organisms and the environment is poor, the outcomes of the model may be sensitive to variation in the strength of stabilising selection, or the additive genetic variance. However, the model assumes a fixed strength of stabilising selection, whereas it might be expected that as the match between a population and a changing environment became poorer, the strength of stabilising selection would increase. Lastly, the estimate of *r_max_* (0.49) employed here may be an underestimate, as this does not include immigrants, which compensate for recruits that have dispersed from the population [28],[36]. In summary, with the other parameters being relatively straightforward to estimate, any systematic bias in parameter estimates is most likely in the direction such that the potential for micro-evolutionary adjustment to climate change is underestimated.

Extrapolating the model using parameters derived from a single great tit population to other bird species suggests that species at the faster end of the life-history continuum would have sufficient evolutionary potential to adapt phenology to a temperature change of the order of 0.030°C y^−1^ (Figures 3a, 3b, and 4). The predicted rates of change for the study area from United Kingdom Climate Projections 2009 (UKCP09) [25] are broadly comparable to predicted rates of global temperature change, as IPCC [37] scenarios predict similar or less temperature change for this century. However, how representative are the parameter estimates derived from this single population for other species and populations? Current knowledge suggests that evolutionary potential of most bird species in terms of phenological adaptation should be broadly similar, since heritability for laying date is not likely to be much greater than the value used here [31], and predictions are not very sensitive to values of *γ* (Figures 3c). While heritability may decrease under adverse environmental conditions [38],[39] the opposite may also apply [40],[41] and at present there is no evidence of climate-related dependence of the heritability of laying date in our study population [40],[42].

Estimates of the optimal phenotypic response to changing environmental conditions (in the present study, the optimal response in laying date to temperature [*B*], as determined from the response of the timing of caterpillar peak abundance to temperature) are not widely available. An estimate of *B* for another very well-studied Dutch population of great tits is lower than the one for our population (−4.01 versus −5.30; [43]), and this is a population for which the phenotypic response of the birds is also lower (see [40] for a comparison), suggesting that |*B−b*| would be larger than in the Wytham population. To the best of our knowledge, there are few comparable estimates from other systems, though see [44]. In general, one can expect that optimal responses are determined by the response of lower trophic levels in the food chain [21],[45]. In that respect, observations for 1,558 largely Northern hemisphere wild plant species suggesting an average advance in spring leafing and flowering of 5–6 d per °C [46], suggest that our estimate of *B* (which is also in units of days per °C) is quite representative of terrestrial systems in the Northern hemisphere. Rates of change in higher trophic levels (i.e., *b*) may be more variable. A large-scale analysis of data from three decades across environments in the UK by Thackeray et al. [3] suggested that while primary producers and consumers have shown broadly comparable rates of advance with climate change, secondary consumers have on average advanced at only about half the rate. Hence, the general expectation might be that *B* and *b* will not be very closely matched, and that a scenario intermediate to the two we modelled (close match between *B* and *b*; no plasticity at all) is most common. It should be noted that our conclusions are drawn from analysis of plasticity in phenology, and given considerable annual variability, phenological traits may have a very high degree of plasticity. Other traits, for instance thermal tolerance, or migration timing, might show less plasticity, but we are not aware of studies of other classes of trait that would support analysis in the framework used here.

Recently a similar approach to calculate the risk of extinction for a Dutch population of great tits yielded a more pessimistic outcome [47]. This is predominantly caused by the combination of lower plasticity, weaker selection, and more extreme climate change scenarios (up to 0.067°C y^−1^) [47]. However, in contrast with our study population, where average offspring recruitment is lower in years with stronger selection on relative laying date [22] and about 13% of annual population growth can be explained by the population's match with the food peak (unpublished data), population growth is hardly affected by the match with the food peak in the Dutch population [30],[47],[48]. This illustrates that even when the match with the food peak is the single most important factor explaining relative fitness, other ecological processes that determine population growth or absolute fitness (e.g., density dependence)—the effects of which on population viability in response to climate change are less straightforward to estimate—can potentially mitigate adverse population effects [30],[48].

In contrast to cases where there is a close tracking of the environment, inability to adjust phenotypically to a gradual shift in optimal timing caused by climate change suggests very high risks of population extinction in species with long generation times (Figures 3b and 4). Such risks could potentially be buffered with higher evolvability, but we are unaware of any evidence for a link between life history and genetic variance. The greater vulnerability of species with slower life histories contrasts with predictions of Morris et al. [49] who suggested longevity should act as a buffer against climatic variability. This raises the question of whether longer-lived species will have already evolved a sufficiently plastic response in timing of reproduction, to variation in temperature, to cope with the relatively fast directional change that is predicted for the future. This is especially relevant as our results show that their long generation time limits their potential to respond with genetic adaptation to climate change.

In conclusion, parameterisation of Chevin et al.'s [13] model with conservative estimates from an extensively studied wild bird population suggests little risk of extinction of that population due to future change in temperature as predicted by climate models. By varying terms in the model we estimated that the absence of phenotypic plasticity would increase the likelihood of population extinction approximately 500-fold. For birds with longer generation times, vulnerability to extinction is considerably higher even for only moderate mismatches of phenotypic plasticity with the rate of environmental change, as they may exhibit insufficient evolutionary potential to adjust to relatively fast change. For those species, phenotypic plasticity in timing of reproduction is likely to be by far the most effective mechanism to cope with constantly increasing temperatures. However, relatively less is known about the determinants and limits on plasticity in such organisms, and increased focus on this area, as well as work on the link between phenotypic plasticity and life history would be very valuable.

## Materials and Methods

### Study Species and General Data Collection

Great tits are small (14–22 g) passerine birds, common in large parts of Europe, Asia, and Northern Africa [50]. They are socially monogamous and breed in cavities, but readily accept nestboxes, if provided. Wytham Woods (Oxfordshire, UK, 51°46′ N 1°20′W) consists of *ca* 385 ha mixed deciduous woodland with an excess of nestboxes (*n* = 1,020) available since 1960. On average 217 nestboxes are occupied annually by great tits [51], although population size has increased in recent decades. Second broods are rare (<3%) and typically excluded from analyses (e.g., [22]). Data collection in the breeding season (April–June) consists of weekly nestbox checks in the laying phase to record first egg date (here referred to as “laying date”) and clutch size. Occupied nestboxes are checked every 2 d around the anticipated hatching date to infer hatching date and allow ringing of nestlings (for future identification) at a standard age of 15 d. At least 5 d later, nestboxes are checked for successful fledging of nestlings. Parents are caught in the nestbox while feeding nestlings, and identified by their ring, or newly ringed if immigrant. Recruits to the natal population are defined as locally hatched birds that were caught as a parent in subsequent years. For analyses in this paper, we use data collected between 1960–2010, as field protocols were standardised over this period.

### The Model

Chevin et al.'s model [13] extends an earlier model by Lynch and Lande [11], by incorporating plasticity in a phenotypic trait (*z*, here first egg-laying date) that mediates adaptation to a changing continuous environmental parameter (*ε*, here temperature). It predicts the maximum rate with which *ε* can change (at a constant rate in time) to allow long-term population persistence, referred to as the critical rate of environmental change (*η*_c_). In the original model [11] *η*_c_ depended only on the phenotypic variance (*σ* ^2^) in *z*, the heritability (*h* ^2^) of *z* (together comprising the additive genetic variance for *z*), the strength of stabilising selection (*γ*, [52]) on *z*, and the maximum intrinsic rate of population growth (*r_max_*). Note that *γ* refers to selection on unstandardised phenotypic variation, assumes the absence of strong directional selection, and a positive value represents stabilising selection, rather than disruptive selection. The extended model also includes the species' generation time (*T*), with *T* being expressed on the same units of time scale as *η*_c_ and *r_max_* (here in years; *r_max_* is measured in years^−1^). Furthermore, it includes the environmental sensitivity of selection (*B*), which reflects how the optimal value of *z* (laying date) depends on *ε* (temperature), and the degree of phenotypic plasticity or reaction norm (*b*), which quantifies the effect of *ε* on *z*, within individuals. Altogether the critical rate of change is modelled as:
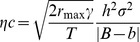
We refer to Chevin et al. [13] for a more detailed description of the model and its rationale.

### Parameterisation of the Model

We used a range of previously published estimates and new analyses to parameterise the model, all of them specific to the Wytham Woods study population. All parameter estimates are listed in Table 1 and, for cases where we used previous estimates from this population, we refer to Figure S1, Text S1, and the specific publication for exact methodological details. Some parameters have been estimated multiple times, and can vary because of different data inclusion criteria, different time spans, different assumptions, or different statistical estimation procedures. In such cases we used the most recent estimate of the respective parameter, as these generally used most data, and employed the most appropriate estimation procedures (see Figure S1 and Text S1 for more discussion).

We estimated the strength of stabilising selection on laying date (*γ*) with the following equation: −(*ω* ^2^+*σ* ^2^)^−1^ = *γ*−*β* ^2^ [53]. The width of the fitness function (*ω*) for laying date was estimated by calculating year-centred laying dates (i.e., subtracting annual average laying date, *n* = 8,646 laying dates in 51 y), categorising them in 10 equally spaced intervals, and calculating the average number of recruits per breeding attempt for each category. A Gaussian function (Figure S1) was fitted to these average numbers and *ω* was estimated as the “standard deviation” of the function (*ω* = 11.62). Phenotypic variance (*σ* ^2^) in laying date was estimated as the average of all annual values (*σ* = 5.39). Since the model by Chevin et al. [13] assumes that the population is initially well adapted, we set the strength of directional selection (*β*) at zero, and calculated *γ* as −0.0061. The assumption of an initially well adapted population, and thus zero directional selection, is required by the model, yet depending on the match with the food peak there can be strong directional phenotypic selection on laying date observed [22]. Since we have no indication that the population is currently poorly adapted, the observed phenotypic selection on laying date may be biased by phenotypic covariance between other aspects of individual quality and laying date (see also Discussion). Using a bootstrapping procedure we estimated the standard error of *γ* as 0.0010. Note that we use the absolute value of *γ* in the model.

A recent study by Husby et al. [40] showed that the average temperature between 15 February and 25 April (here referred to as “spring temperature”) is the best predictor of average annual laying date; we thus used the individual response in laying date to this environmental variable as an estimate of phenotypic plasticity, and the response in the date of standardised caterpillar abundance as an estimate of environmental sensitivity (see details below). A similar exercise to that of Husby et al. [40] had been performed earlier, but based on a longer time series and a slightly different environmental variable, i.e., “warmth sum” (the sum of the daily maximum temperatures between 1 March and 25 April, [22]). We chose to conduct analyses with the average temperature, as used by Husby et al. [40], to permit more straightforward comparison between the modelled critical rate of environmental change and predictions about future climate change; see [22] for detailed information on how great tit laying date and peak caterpillar abundance date have changed over time.

We used the daily average of minimum and maximum temperatures (in °C) that were collected by the Radcliffe Observatory in Oxford, 5 km east of Wytham Woods, for our measure of spring temperature. The date by which 50% of the seasonal total of winter moth caterpillars (*Operopthera brumata* larvae, the main source of food for great tit nestlings; [54]–[56]) had descended from trees to pupate on the ground (here referred to as “caterpillar half-fall date”) was recorded in Wytham Woods in the majority of years from 1961 onwards (*n* = 43), and gives a good indication of the timing of the peak in caterpillar biomass (see [22] for more details). Given a fixed period between great tit laying date and peak offspring food demand, this serves as a proxy for the optimal response in laying date to spring temperature [22]. Hence, environmental sensitivity of selection (*B*) was accordingly calculated as the slope of the linear function of caterpillar half-fall date in response to spring temperature.

Phenotypic plasticity, or the average within-individual response in laying date to changes in spring temperature, was calculated from a dataset restricted to females that bred at least twice (*n* = 4,742 reproductive attempts of 1,874 females, in 51 y). The within-individual slope was calculated by using the difference between the spring temperature a female experienced before a specific reproductive attempt with the average of the spring temperatures a female experienced before all her reproductive attempts, as explanatory variable in a model on laying date (following [57]). In the model we also included the average of the spring temperatures a female experienced before all her reproductive attempts as explanatory variable, to account for potential micro-evolution or selective (dis)appearance of individuals with higher, or lower, average spring temperature experience. Female identity, year, and sector of the wood (Wytham Woods consists of nine different sectors with different vegetation types and management regimes, see [58]) were included as random effects, to correct for an uneven distribution of repeated measures of individuals, inter-annual variation (not due to spring temperature) and environmental heterogeneity, respectively. Models were fitted with a normal error distribution and a Markov Chain Monte Carlo estimation algorithm with 100,000 iterations, using MLwiN version 2.02 [59],[60]. Significance of explanatory terms was determined using the Wald statistic, which approximates the χ^2^ distribution.

We used projections from the United Kingdom Climate Projections 2009 (UKCP09, [25]) to compare our results against the predicted rate of average temperature change for the Wytham Woods area. To this end, we used the average temperature change predictions for the 25-km grid box that contained Wytham Woods (number 1,547) for the 2070–2099 time period, under the low, medium, and high emissions scenario, for the months February, March, and April. We weighted the predictions per month according to their number of days contained in our measure of spring temperature (see above). To calculate an annual rate of change we used the midpoint of 2070–2099 relative to the midpoint of the baseline period (1961–1990). This resulted in a predicted rate of increase of spring temperature of 0.021, 0.025, and 0.030°C y^−1^ for the low, medium, and high emissions scenario, respectively.

## Supporting Information

Figure S1 **Mean number of recruits with respect to mean-centered laying date, with fitted Gaussian function used to estimate stabilising selection on laying date for Chevin's model.**
(TIFF)

Text S1 **Parameter estimation for the Chevin et al. model.**
(DOCX)

## Abbreviations

SE: standard error
