# Supplementary figures and images for: Quantitative Assessment of the Importance of Phenotypic Plasticity in Adaptation to Climate Change in Wild Bird Populations
Source: PLoS Biol. 2013 Jul 9;11(7):e1001605. doi: 10.1371/journal.pbio.1001605 (PMC3706305; doi:10.1371/journal.pbio.1001605)

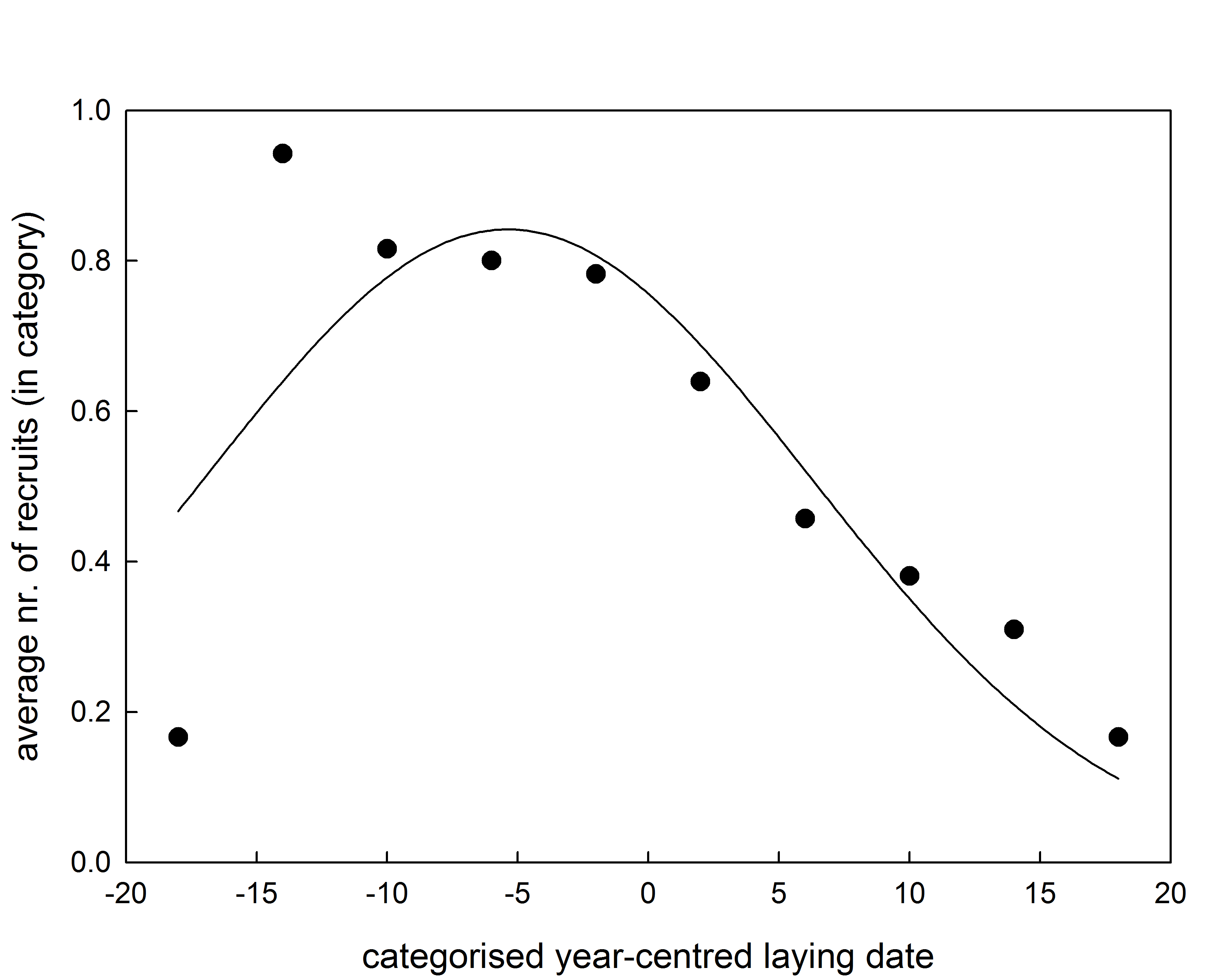

Supplement: Figure S1 — Mean number of recruits with respect to mean-centered laying date, with fitted Gaussian function used to estimate stabilising selection on laying date for Chevin's model. (TIFF) [file pbio.1001605.s001.tiff]
