# Supplementary material for: Quantitative Assessment of the Importance of Phenotypic Plasticity in Adaptation to Climate Change in Wild Bird Populations
Source: PLoS Biol. 2013 Jul 9;11(7):e1001605. doi: 10.1371/journal.pbio.1001605 (PMC3706305; doi:10.1371/journal.pbio.1001605)
Supplement: Text S1 — Parameter estimation for the Chevin et al. model. (DOCX) [file pbio.1001605.s002.docx]

*Supplementary material accompanying*

**Quantitative assessment of the importance of phenotypic plasticity in adaptation to climate change in wild bird populations**

Oscar Vedder, Sandra Bouwhuis & Ben C Sheldon

**Parameter estimation for the Chevin et al. model**

Chevin et al.’s model [1] involves the estimation of six parameters that are then fitted to a mechanistic model to predict the critical rate of environmental change, beyond which a population is not expected to be able to keep track with a changing environment.

These parameters are as follows:

(1) *σ^2^h^2^* Additive genetic variance of the trait

(2) *γ* Strength of stabilising selection on the trait

(3) *T* Generation time

(4) *r_max_* Maximum intrinsic rate of population growth

(5) *B* Environmental sensitivity of selection: the rate at which the trait optimum varies with the environment

(6) *b* Phenotypic plasticity of the trait

In the present case we applied this model to a long term study of great tits from Wytham Woods, near Oxford, where the environmental sensitivity of selection is defined as the rate at which a standard measure of peak abundance of caterpillars on which birds feed their nestlings changes with the environment. Hence, the assumption here is that there is a population-specific optimum each year, which can be defined by the timing of the food peak. With this assumption, we can model the extent to which selection and phenotypic plasticity in first egg date (laying date) enables the population to remain viable as the environment changes. We chose to analyse first egg-date as the trait determining the population’s ability to match its environment because (i) this is a well-defined phenotype with a clear connection to the annual cycle, (ii) it has been the subject of considerable study in this and other populations of birds and (iii) it largely defines the timing of subsequent events in the reproductive cycle. To some extent, females are able to adjust the hatching date of their clutch after egg laying, and either accelerate or retard hatching by either beginning incubation before clutch completion, or delaying incubation for some days after clutch completion, respectively [e.g. 2,3]. However, we chose not to use hatch date as our phenotypic measure of environment matching for three reasons. First, it has not been recorded as part of our standard field protocols, and its use thus requires estimating an inferred hatching date, based on the size of nestlings. Since growth rates may be variable, or estimation imprecise, we preferred to focus on data we had directly collected. Second, the occurrence of hatching asynchrony (broods may hatch over 48h or more) makes hatching date harder to define as a phenotype. Third, because a proportion of clutches that are laid do not hatch, there is the possibility that timing-related selection is under-estimated if only the sample of nests that produce hatched nestlings is used.

Of the parameters in the model, some have been estimated previously, sometimes several times, using a range of methods. We expand here on the methodologies used by these different studies and explain the choice of method we followed here.

1. *Additive Genetic Variance of Laying Date*

Four studies have reported five estimates of the heritability of laying date from great tits in this study population using a range of methods and assumptions; these were summarised by Liedvogel et al. [4], together with heritability estimates of laying date in other great tit populations (and in all other bird species for which this had been estimated at the time). McCleery et al. [5] used an animal model approach with a limited number of fixed and random effects and estimated *h^2^* for laying date of 0.16. Garant et al. [6] used an animal model, again with limited fixed and random effect structure, to estimate the heritability of laying date in two periods (pre 1989 and post 1989, in a paper testing whether quantitative genetic parameters differed between two period with contrasting patterns of change in spring climate. That study estimated *h^2^* = 0.16 and *h^2^* = 0.09 for 1965-1988 and 1989-2004 respectively. Van der Jeugd & McCleery [7] used mother-daughter regression but, importantly, showed that the heritability estimate returned by this method depended on the distance that the daughter had dispersed from the mother, with heritabilities higher for daughters that settled close to their mother, which they interpreted as being due to spatial autocorrelation in the environment leading to inflated heritabilities. Although the estimates are probably not comparable with those derived from an animal model (a general observation is that parent-offspring heritablities are higher than those that estimated in the mixed model framework on an animal model [8]), this paper established that controlling for similarity in the environments experienced by relatives may be important in estimating heritabilities. Finally, Liedvogel et al. [4] estimated heritability of laying date fitting several fixed effects that correct for known environmental influences on laying date (altitude, conspecific density) as well as random effects that would not contribute to an evolutionary response (nestbox, permanent environment effects for male and female) and obtained a lower estimate for heritability of laying date of *h^2^* = 0.03 (±0.01 SE). We used the additive genetic variance from this analysis in the current paper, because we considered this the most appropriate model in terms of accounting for covariance in the environment between parents and offspring.

*(2) Strength of Stabilising Selection on Laying Date*

As described in the main text, we estimated the average strength of stabilising selection on laying date for the population from the width of the fitness function fitted to non-standardised (though year mean-centred) data over all 51 years (see fig 1) , with the standard error for this estimate obtained by bootstrapping. Two previous studies have reported estimates of non-linear selection on laying date from this population. Garant et al. [9] reported a non-linear selection gradient (on standardised data) on laying date (in a model that included selection on clutch size and egg mass) of *γ’* = -0.027 ± 0.014, when analysing data from 1965-2000, suggesting that selection is stabilising overall. Charmantier et al. [10] analysed annual variation in selection, and showed that the parameter estimate for non-linear selection from a model with linear and quadratic terms was related to the degree of population-wide synchrony between great tits and caterpillars (fig 2b in that paper). In effect, in years when a large part of the population breeds in synchrony with the food peak, selection is more strongly stabilising. In contrast, in years in which the population is poorly synchronised, the fitness function relating laying date to recruitment success is concave (although not disruptive; note that a positive quadratic coefficient in a selection analysis does not necessarily indicate disruptive selection). This paper is interesting in the present context, as it suggests that the form of selection may depend on the match between birds and their environment (i.e. covariance between *γ* and |*B*-*b*|); further development of the theoretical framework used here might explore the consequences of non-independence of the parameters.

As also discussed in the main text, the strength of stabilising selection may be underestimated because, in such analyses, phenology is defined relative to the population mean. However, if, as seems likely, environments vary spatially in their optimal phenology, and organisms are selected to match their local timing (in the case of birds, this might occur because they are central place foragers and selected to minimise travel-time when making repeated foraging trips), then a population-level definition of relative phenotype has the effect of systematically displacing locally-adapted individuals from their local optimum. Consequently, individuals that time their reproduction, and thus maximise their fitness in relation to their local phenological landscape are, if defined relative to the population mean, assessed on the wrong scale. Future work in this area should address the relative importance of local and population-level scales in determining optimal timing.

Fig. 1 Gaussian function fitted to the average number of recruits for 10 equally-spaced year-centred laying date intervals. The width of the fitness function (*ω*) was estimated as the ‘standard deviation’ of the Gaussian function (*ω* = 11.62)

*(3) Generation Time*

We used a previous estimate of generation time, defined simply as the average age (± SE) at which females reproduced as reported in [11]. This is based on 7341 breeding records of 4935 females of known age. Since reproductive senescence occurs in this population, with recruitment success decreasing past the peak reproductive output (c. 2.8 years), this figure could be slightly revised downwards if one accounted for the age-specific differences in reproductive value, but the effect will be quite small (e.g. see [12]). The difference with a previous estimate of Wytham Woods’ great tit generation time, using a lifetable approach [13], was negligible (1.80 vs 1.81).

*(4) Maximum Intrinsic Rate of Population Increase*

We used the value of *r_max_* in years^-1^ (0.49) as estimated in [13] based on lifetable data of Wytham Woods’ great tits, presented in [14]. In detail; maximum values of survival and fecundity were used to calculate the maximum annual growth rate (*λ_max_*) as the dominant eigenvalue of a Leslie matrix model, accounting for age dependence in demographic parameters [15], with the ULM program [16]. *r_max_* was subsequently calculated as ln[*λ_max_*], following [17].

*(5) Environmental sensitivity of selection*

As described in detail in the main paper, this was estimated simply from the slope of the regression of caterpillar half-fall date in each year on the mean temperature from 15 February to 25 April. We used the same interval to define the environment for both caterpillar timing and bird laying date because we wanted to be able to compare each on a common scale. Previous studies (e.g. [10]) have reported the relationship between caterpillar timing and spring temperature, but these are not directly comparable because they did not include data from very recent years, and used different temperature indices (see main text).

*(6) Phenotypic plasticity of the trait*

Plasticity of laying date was estimated, as described in detail in the main text, by fitting a model that estimates the within-individual effect of temperature on laying date while also estimating the between individual effect. The approach is similar to that used in [10] except that again, a broader range of years (including recent data), and a different temperature index were used in this study (again, see main text for more details).

**Literature cited**

1. Chevin LM, Lande R, Mace GM (2010) Adaptation, plasticity, and extinction in a changing environment: towards a predictive theory. PloS Biol 8: e1000357.
2. Cresswell W, McCleery R (2001) How great tits maintain synchronization of their hatch date with food supply in response to long-term variability in temperature. J Anim Ecol 72: 356-366.
3. Vedder O (2012) Individual birds advance offspring hatching in response to increased temperature after the start of laying. Oecologia 170: 619-628.
4. Liedvogel M, Cornwallis CK, Sheldon BC (2012) Integrating candidate gene and quantitative genetic approaches to understand variation in timing of breeding in wild tit populations. J Evol Biol 25: 813-823.
5. McCleery RH, Pettifor RA, Armbruster P, Meyer K, Sheldon BC, Perrins CM (2004) Components of variance underlying fitness in a natural population of the great tit *Parus major*. Am Nat 164: E62-E72.
6. Garant D, Kruuk LEB, Hadfield JD, Sheldon BC (2008). Stability of genetic variance and covariance for reproductive characters in the face of climate change in a wild bird population. Mol Ecol17: 179-188*.*
7. van der Jeugd HP, McCleery R (2002) Effects of spatial autocorrelation, natal philopatry and phenotypic plasticity on the heritability of laying date. J Evol Biol 15: 380-387.
8. Kruuk LEB, Slate J, Wilson AJ (2008) New answers for old questions: the evolutionary quantitative genetics of wild animal populations. Annual Reviews of Ecology, EvoluSystem 39: 525-548.
9. Garant D, Kruuk LEB, McCleery RH, Sheldon BC (2007) The effects of environmental heterogeneity on multivariate selection on reproductive traits in female great tits. Evolution 61: 1546-1559.
10. Charmantier A, McCleery RH, Cole LR, Perrins C, Kruuk LE, Sheldon BC (2008) Adaptive phenotypic plasticity in response to climate change in a wild bird population. Science 320: 800-803.
11. Bouwhuis S, Sheldon BC, Verhulst S, Charmantier A (2009) Great tits growing old: selective disappearance and the partitioning of senescence to stages within the breeding cycle. Proc R Soc Lond B 276: 2769-2777.
12. Bouwhuis S, Choquet R, Sheldon BC, Verhulst S (2012). The form and fitness cost of senescence: age-specific recapture, survival, reproduction and reproductive value in a wild bird population. Ame Nat 179: E15-E27*.*
13. Niel C, Lebreton JD (2005) Using demographic invariants to detect overharvested bird populations from incomplete data. Conserv Biol 19: 826-835.
14. McCleery RH, Perrins CM (1989) Lifetime reproductive success of the great tit, *Parus major*. In: Newton I, editor. Lifetime reproduction in birds. San Diego, USA, Academic press. pp. 35-55.
15. Caswell H (2000) Matrix population models. 2^nd^ edition. Sunderland, Massachusetts, USA, Sinauer.
16. Legendre S, Clobert J (1995) ULM, a software for conservation and evolutionary biologists. J Appl Statist 22: 817-834.
17. Odum EP (1971) Fundamentals of Ecology. Philadelphia, USA, Saunders.
